# The Role of JAK Inhibitors in Pediatric Lichen Sclerosus et Atrophicus: A Case Report

**DOI:** 10.7759/cureus.85922

**Published:** 2025-06-13

**Authors:** Shivangi Sharma, Akanksha Bandhade, Mrityunjay K Singh, Vinod Koshley, Sayali P Gongale

**Affiliations:** 1 Dermatology, Pandit Jawaharlal Nehru Memorial Medical College and Dr. Bhimrao Ambedkar Memorial Hospital, Raipur, IND

**Keywords:** case report, extragenital, lichen sclerosus, oral tofacitinib, pediatric age group

## Abstract

Lichen sclerosus (LS) is an uncommon mucocutaneous condition that primarily affects the genitoanal region and is characterized by white, porcelain-like sclerotic lesions. Although its incidence peaks after menopause, LS can also occur in children, affecting both the genital and extragenital areas. We present the case of a pediatric patient with both genital and extragenital LS who responded well to oral tofacitinib therapy. This case highlights that oral tofacitinib is a safe and effective treatment option for lichen sclerosus et atrophicus (LSA) in the pediatric population.

## Introduction

Lichen sclerosus et atrophicus (LSA) is a chronic inflammatory dermatosis affecting both the dermis and epidermis, with an unknown etiology [1]. It typically presents as white, opalescent papules that may coalesce into parchment-like plaques. LSA predominantly affects the anogenital region in 83%-98% of cases, while 15%-20% involves extragenital areas [2]. The condition is more common in prepubertal, perimenopausal, and postmenopausal females, primarily affecting the vulva, perineum, and perianal skin. Extragenital LSA is often asymptomatic but may cause pruritus and commonly appears on the neck, shoulders, and upper torso. Here, we present a pediatric case of both genital and extragenital LSA that responded well to oral tofacitinib therapy.

## Case presentation

A 7-year-old female patient presented to the dermatology outpatient department with multiple ivory-white macules distributed across her body, associated with pruritus for one year. She also reported progressive difficulty in defecation and constipation. The patient had no personal or family history of autoimmune diseases or similar dermatological conditions and was otherwise healthy.

She had previously been treated at a peripheral healthcare center with topical clobetasol for two months and tacrolimus for four months. These treatments were administered without structured follow-up, and due to lack of improvement, they were discontinued by the patient’s family. No photographic documentation was available from this treatment period. Six months later, the patient experienced worsening constipation, an increase in the size of the genital lesion, and the appearance of new whitish patches on the trunk and lower limbs, prompting her visit to our clinic.

**Figure 1:**
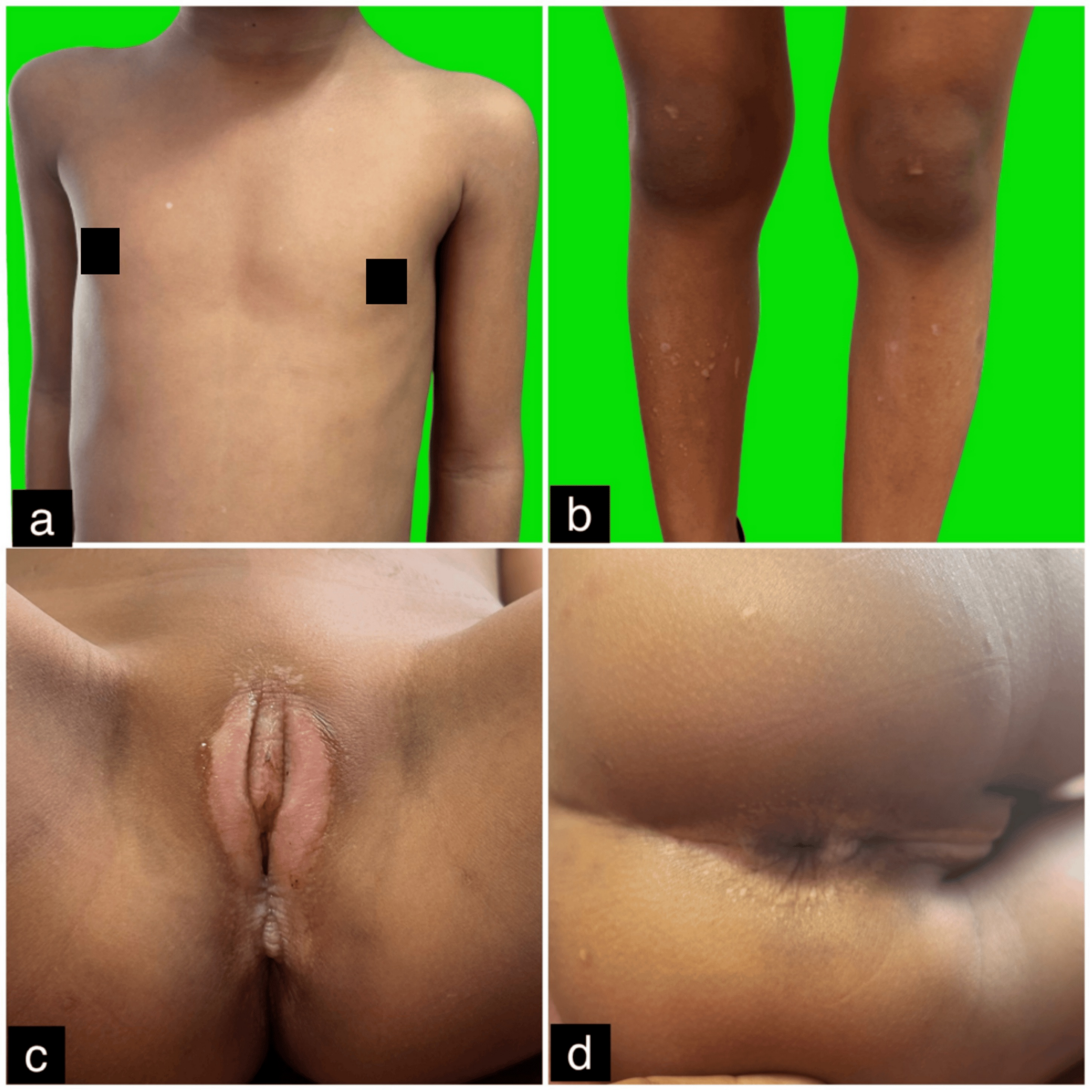
(a-d) Clinical photographs revealing multiple small white macules (about 0.5-1 cm in size) on the chest and bilateral legs, along with a single large atrophic patch involving the bilateral labia majora and vestibule, extending to the perianal skin.

**Figure 2:**
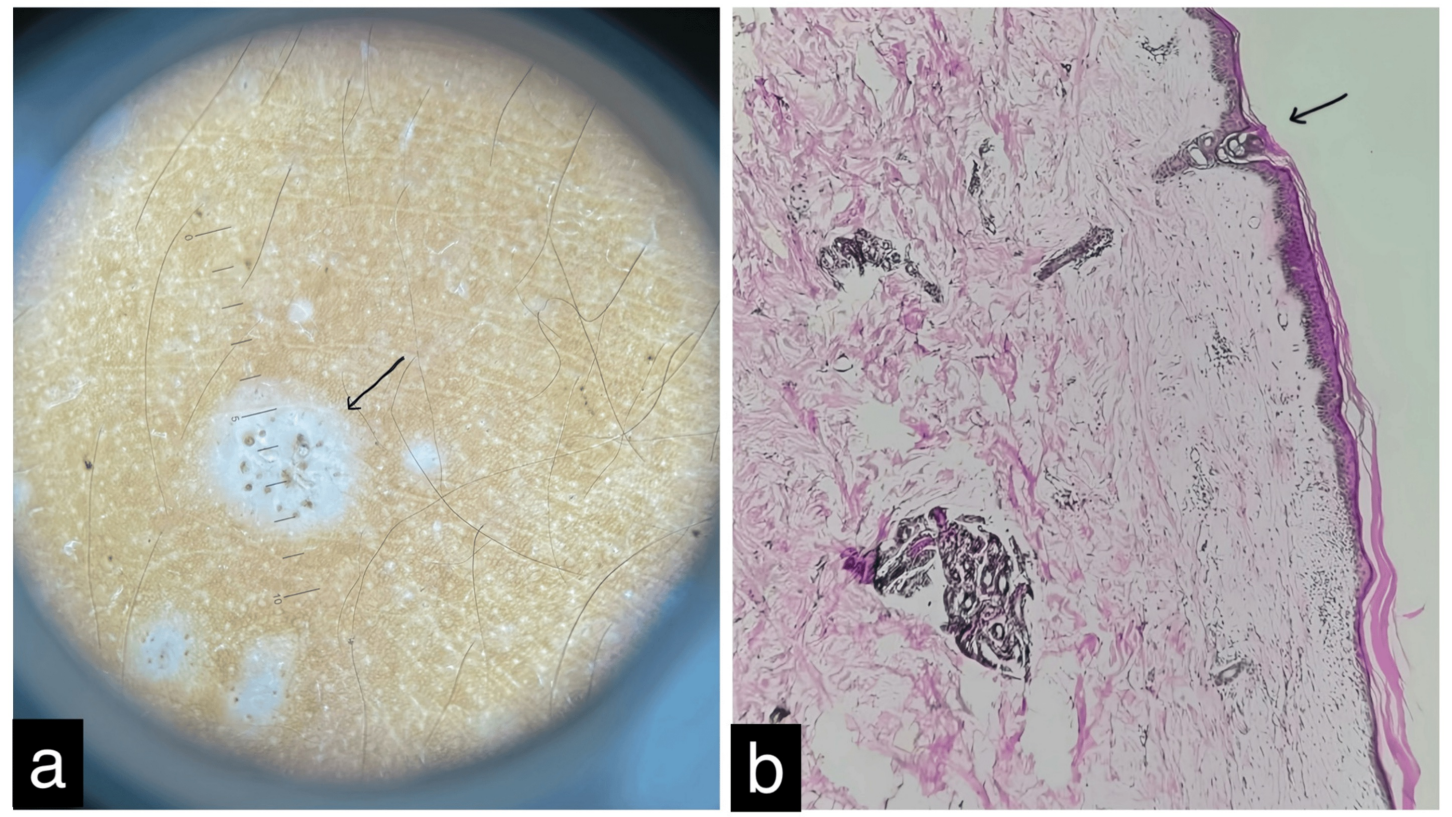
(a) Dermoscopy of the macules revealing homogenous, structureless white areas with follicular plugs. (b) Histopathology of a leg lesion showing mild perivascular and periadnexal lymphocytic cell infiltrates in the deep dermis, with sparse lymphocytic infiltrates in the superficial dermis and homogenization of dermal collagen. The overlying epithelium exhibited marked atrophy with follicular plugging (magnification 40x).

On physical examination, multiple white macules (0.5-1 cm) were observed on the chest and bilateral legs, along with a large atrophic patch involving the bilateral labia majora, vestibule, and perianal region (Figures 1a-1d). The genital mucosa was involved, while the oral mucosa appeared normal. No lymphadenopathy was present, and systemic examination was unremarkable. A clinical diagnosis of genital and extragenital LSA was made. Dermoscopy revealed homogeneous, structureless white areas with follicular plugs (Figure 2a). The differential diagnoses considered included LS, guttate vitiligo, and guttate morphea. Routine blood tests, peripheral smear, urine analysis, chest X-ray, viral markers, ECG, and Bacille Calmette-Guérin (BCG) skin prick test were all within normal limits. A skin biopsy from a lesion on the leg showed mild perivascular and periadnexal lymphocytic infiltrates in the deep dermis, sparse lymphocytic infiltrates in the superficial dermis with homogenization of dermal collagen, and marked epidermal atrophy with follicular plugging (Figure 2b). These histopathological findings confirmed the diagnosis of genital and extragenital LSA. 

**Figure 3:**
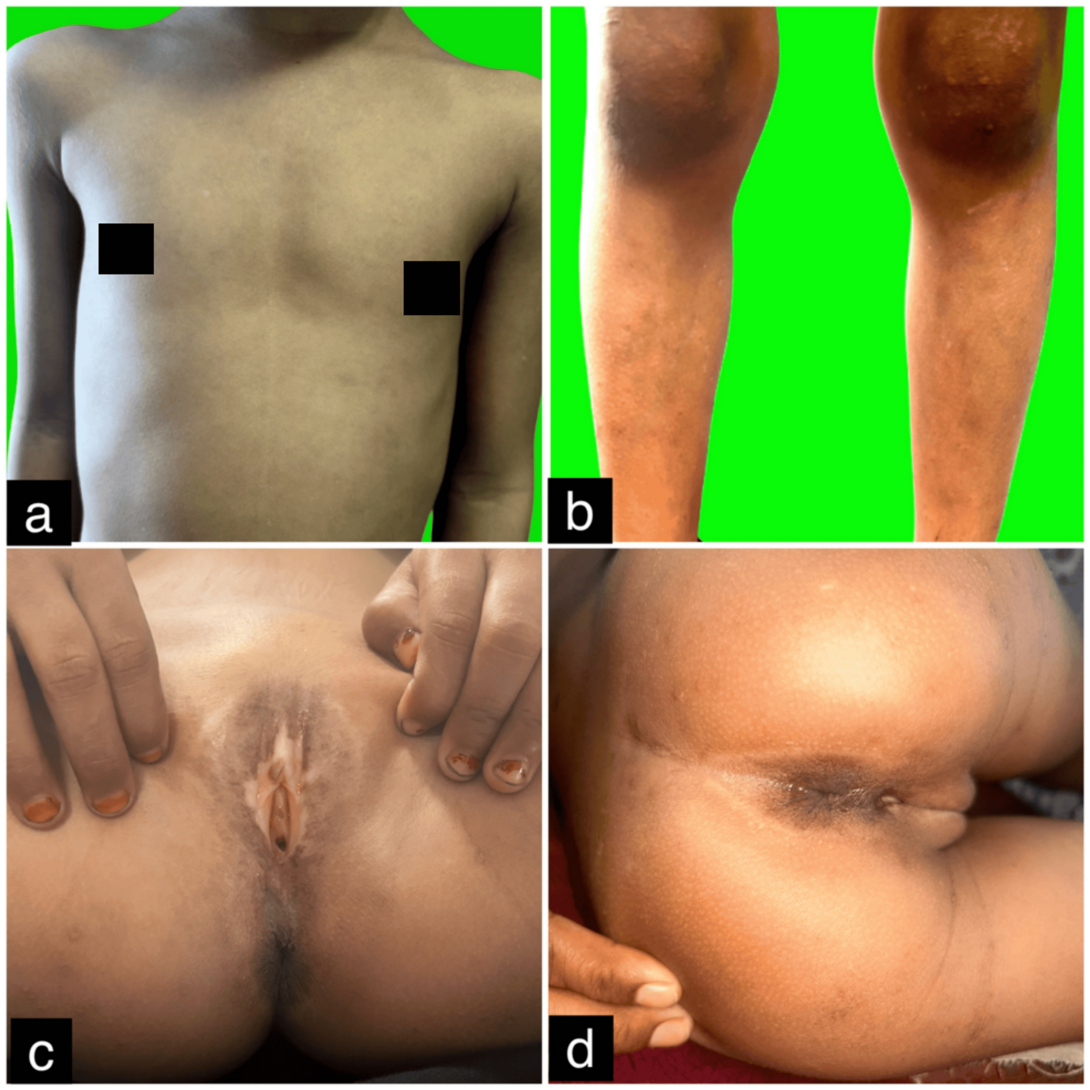
(a-d) Follow-up images at six months showing significant reduction in pruritus. The lesions progressed from depigmented to hypopigmented, with some areas demonstrating complete repigmentation.

**Figure 4:**
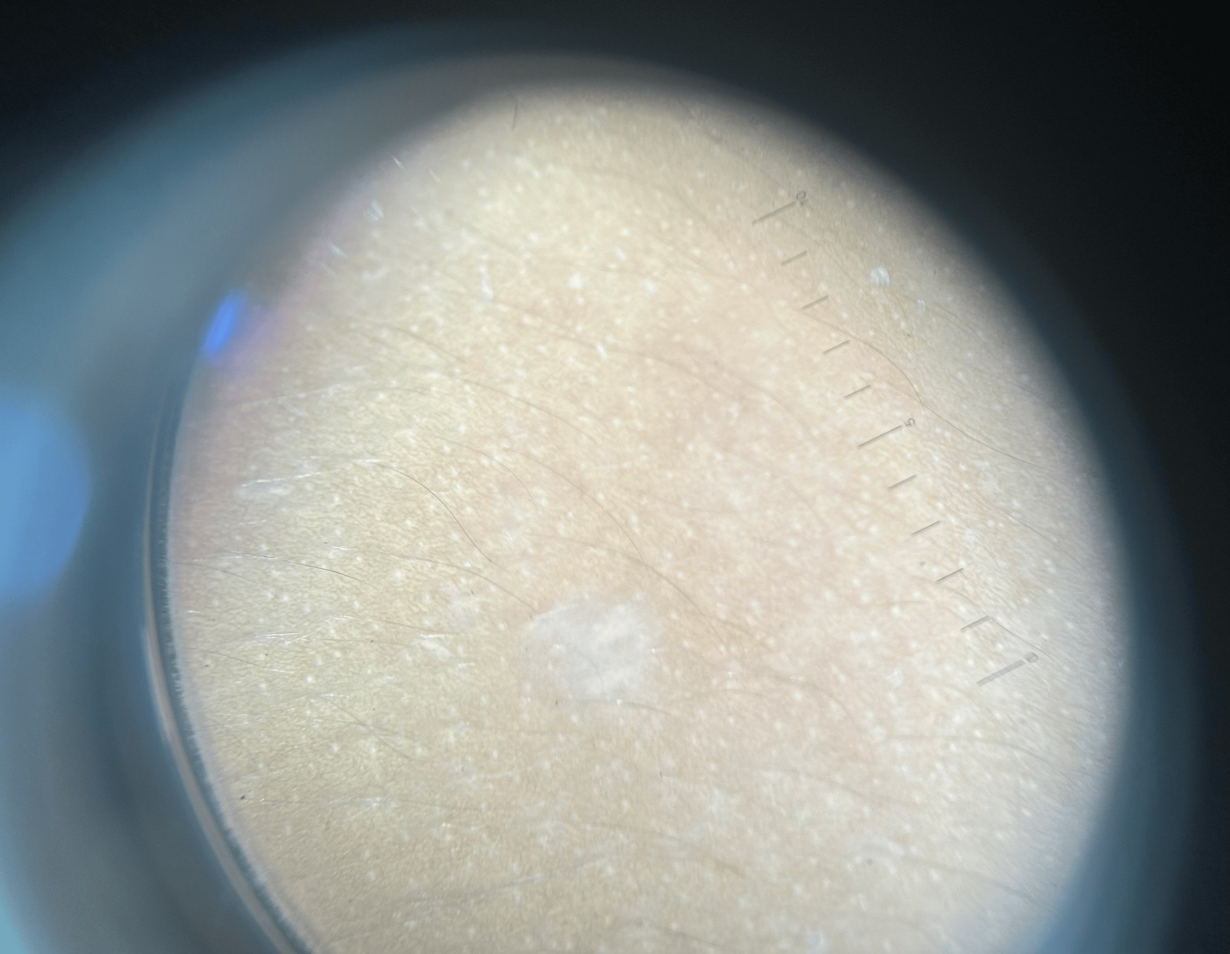
Dermoscopy of the chest lesion after six months of oral tofacitinib treatment, showing the transformation from a depigmented to a hypopigmented appearance, with resolution of follicular plugging.

Given the parental refusal of intralesional steroids and concerns about pain and the potential systemic toxicity of other options such as acitretin and methotrexate, and considering the shared involvement of the JAK/STAT pathway in both LSA and vitiligo, the patient was started on oral tofacitinib 5 mg once daily, following a short one-week course of oral steroids. She was then maintained on oral tofacitinib 5 mg daily and syrup cetirizine for pruritus, both prescribed according to her body weight. A pediatric surgery consultation for constipation revealed no significant atrophy of the anal mucosa, and she was prescribed syrup lactulose. At the 6-month follow-up, her pruritus had significantly improved, and the lesions had evolved from depigmented to hypopigmented, with some areas showing complete repigmentation. Her constipation had also completely resolved. Follow-up images demonstrated marked clinical improvement (Figures 3a-3d). The patient remains under close follow-up. Dermoscopy of the chest lesion after six months of oral tofacitinib therapy demonstrated a transformation from depigmentation to hypopigmentation, accompanied by resolution of follicular plugging (Figure 4).

## Discussion

LS is relatively rare in children, with one study reporting a prevalence of at least 1 in 900 [3]. It most commonly affects the anogenital region, while extragenital involvement is even less frequent. The condition is observed about 10 times more often in females than in males [4].

The development of vulvar LS (VLS) is complex and multifactorial, likely resulting from a combination of genetic susceptibility, immune system dysregulation, and abnormal collagen metabolism, potentially triggered by environmental factors. An autoimmune mechanism is strongly implicated, as many patients with VLS also present with comorbid autoimmune disorders such as thyroiditis, vitiligo, or alopecia areata. The disease is characterized by a predominant Th1 immune response, with elevated levels of proinflammatory cytokines, including IL-1, IL-7, IL-15, IFN-γ, and TNF-α [5,6]. 

Tofacitinib, a JAK inhibitor that primarily targets JAK1/3, has been successfully used in the treatment of pediatric vitiligo by disrupting key signaling pathways involved in autoimmunity [7]. Since both LS and vitiligo share features such as depigmentation and may co-occur, a potential overlap in their underlying pathogenetic mechanisms has been proposed [8]. This connection may be explained by epitope spreading, a process in which tissue damage from an autoimmune or inflammatory process exposes previously sequestered antigens, subsequently triggering a secondary immune response. In vitiligo, the IFN-γ-JAK/STAT-CXCL10 axis plays a crucial role [9]. Notably, similar proinflammatory mediators (including IFN-γ, CXCR3, CXCL9, CXCL10, and CXCL11) are markedly increased in LS. By inhibiting JAK1/3, tofacitinib may reduce IFN-γ and CXCL10 expression, attenuate cytotoxic T-cell infiltration, and ultimately facilitate repigmentation, as demonstrated in prior studies [10].

In this report, we describe a pediatric case involving both genital and extragenital LS. Although conventional therapies for LS include topical and intralesional corticosteroids, retinoids, phototherapy, estrogen, vitamins, topical tacrolimus, and even surgical approaches [11], several of these options were deliberately avoided in this case due to patient-specific and treatment-related considerations. Oral retinoids, occasionally employed in refractory LS, raise significant concerns in pediatric populations due to potential adverse effects such as skeletal toxicity, hepatotoxicity, and hyperlipidemia [12]. Similarly, topical retinoids, while effective in certain dermatoses, are poorly tolerated in sensitive anogenital and perianal areas, often causing local irritation, erythema, and symptom exacerbation, factors particularly problematic in children [13]. Phototherapy, though beneficial in select chronic skin conditions, was deemed unsuitable because of the impracticality of frequent hospital visits, limited efficacy on mucosal lesions, and long-term risks including photoaging and carcinogenesis [14]. Furthermore, our patient had a documented history of poor adherence to topical regimens, which further limited the utility of topically intensive treatment approaches. Given these factors, oral tofacitinib was selected as a therapeutic alternative and yielded promising clinical outcomes. Early and effective management of LS is critical to prevent long-term complications, including squamous cell carcinoma, secondary infections, and stenosis of the anal and genital orifices.

## Conclusions

This case highlights the promising role of JAK inhibitors, specifically oral tofacitinib, in the management of pediatric LSA involving both genital and extragenital regions. In patients unresponsive to conventional therapies, tofacitinib may serve as a viable and effective alternative, owing to its targeted mechanism of action and its ability to modulate key inflammatory cytokine pathways implicated in the disease. Early initiation of JAK inhibitor therapy may not only improve clinical outcomes but also reduce the risk of long-term complications such as scarring, stenosis, and associated psychosocial distress. However, further large-scale studies are warranted to establish the safety profiles and long-term efficacy of JAK inhibitors in the pediatric population.
